# A Comprehensive Profile of Heart Failure Patients Across Ejection Fraction Subtypes: Insights from a Single-Center Retrospective Cohort Study

**DOI:** 10.3390/medicina61091533

**Published:** 2025-08-27

**Authors:** Austėja Kaunaitė, Austė Markevičiūtė, Vitas Barauskas, Edita Jankauskienė, Vytautas Zabiela, Diana Žaliaduonytė

**Affiliations:** 1 Faculty of Medicine, Medical Academy, Lithuanian University of Health Sciences, 44307 Kaunas, Lithuania; austeja.kaunaite@stud.lsmu.lt (A.K.); vitas.barauskas@stud.lsmu.lt (V.B.); 2 Department of Cardiology, Lithuanian University of Health Sciences, Kaunas Clinics, 44307 Kaunas, Lithuania; edita.jankauskiene@kaunoklinikos.lt (E.J.); vytautas.zabiela@kaunoklinikos.lt (V.Z.); 3 Department of Cardiology, Lithuanian University of Health Sciences, 44307 Kaunas, Lithuania; diana.zaliaduonyte@lsmu.lt; 4 Kaunas Region Society of Cardiology, 50009 Kaunas, Lithuania; 5 Department of Cardiology, Lithuanian University of Health Sciences, Kaunas Hospital, 45130 Kaunas, Lithuania

**Keywords:** echocardiography, heart failure, HFpEF, HFmrEF, HFrEF

## Abstract

*Background and Objectives*: Heart failure (HF) is a complex clinical syndrome with a high prevalence and significant morbidity. Classification of HF into preserved (HFpEF), mildly reduced (HFmrEF), and reduced ejection fraction (HFrEF) groups helps improve patient stratification and treatment. This study aimed to compare clinical, laboratory, echocardiographic, and electrocardiographic characteristics between different HF ejection fraction groups in a single-center patient cohort. *Materials and Methods*: A retrospective analysis of 1144 patients hospitalized with HF between 2022 and 2023 was performed. Patients were divided into three groups based on left ventricular ejection fraction. *Results*: HFrEF patients were predominantly male and younger, while HFpEF and HFmrEF groups had a higher proportion of older females. Atrial fibrillation and arterial hypertension were more common in HFpEF, while HFrEF patients showed more significant ventricular and atrial remodeling, lower tricuspid annular plane systolic excursion (TAPSE) values, and higher N-terminal pro-B-type natriuretic peptide (NT-proBNP) levels. No significant difference in in-hospital outcomes was found between HF groups. *Conclusions*: HF subtypes demonstrate distinct clinical and structural profiles, supporting the need for phenotype-based diagnostic and therapeutic approaches.

## 1. Introduction

Heart failure (HF) is a clinical syndrome that results from structural or functional damage to the heart, impairing the ability of the ventricles to fill with blood during diastole or to push blood out of the heart during systole, all of which lead to symptoms and clinical signs of cardiac dysfunction [1]. It is estimated that there are more than 60 million people with HF worldwide, equivalent to between 1% and 3% of the world’s entire population [2]. Due to its high prevalence, morbidity, and mortality worldwide, it essential to diagnose and manage HF effectively in order to prevent recurrent hospital admissions, reduce complications, and improve patient outcomes [1].

The incidence and prevalence of HF is increasing due to a growing elderly population and improved survival rates after acute ischemic events [3]. In response, healthcare professionals are carrying out an increasing number of studies to find out not only how to reduce mortality from HF, but also how to slow down the progression of the disease, reduce the symptoms, and increase functional capacity in order to improve the quality of life of people with HF [4].

Patients with HF are divided into three groups according to their left ventricular ejection fraction (LVEF): reduced ≤ 40% (HFrEF), mildly reduced 41–49% (HFmrEF), and preserved ≥ 50% (HFpEF). This classification enables a personalized therapeutic approach and allows clinicians to better understand disease progression [5]. However, despite guideline-based definitions, HF subtypes often show overlapping features and distinct clinical, structural, and laboratory profiles, highlighting the importance of comprehensive phenotyping.

Previous studies have shown that post-treatment care by specialists improves patient survival, reduces hospital admissions, and lowers treatment costs [6]. Furthermore, new, long-term patient follow-up and disease management strategies are also being implemented with the goal to educate patients about HF by teaching them self-care and promoting lifestyle changes [7].

While large, multinational registries have examined HF patients by ejection fraction categories before, single-center data may reflect distinct local population characteristics, clinical practice patterns, and comorbidity profiles. Therefore, the main aim of our retrospective cohort study was to provide a detailed characterization of clinical, laboratory, electrocardiographic, and echocardiographic features of HF patients from a single tertiary care center, comparing patient groups classified by ejection fraction.

## 2. Materials and Methods

This retrospective study included data from 1144 patients with HF hospitalized at the Cardiology Department of Kaunas Hospital of Lithuanian University of Health Sciences between 2022 and 2023. The inclusion criteria for this research were as follows: (1) hospitalization in the Cardiology Department during the period of 2022–2023 and (2) a confirmed diagnosis of chronic heart failure, based on ICD-10 codes I50 or I11.0. Data was collected from medical records (sociodemographic characteristics, clinical presentation of HF, and biochemical, electrocardiographic, and echocardiographic findings). Echocardiographic evaluation was performed within the first 48 h of hospitalization. All subjects were divided into three groups according to LVEF: HfpEF-EF ≥ 50% (*n* = 362), HfmrEF-EF 41–49% (*n* = 263), and HfrEF-EF ≤ 40% (*n* = 519). Permission No.: BEC2-605 from the Bioethics Centre of Lithuanian University of Health Sciences was obtained on 8 May 2024 for this study.

Statistical data analysis was performed using IBM SPSS Statistics 29.0 software. Differences in the values of qualitative attributes between the groups were tested using the Chi-square test. The normality of the data was assessed by the Kolmogorov–Smirnov test. The Kruskal–Wallis and ANOVA tests were used. Data differences were considered statistically significant at *p* < 0.05.

## 3. Results

### 3.1. Clinical and Demographic Characteristics

To better analyze and evaluate the data of patients with HF and to see the differences between LVEF groups, patients were divided into three groups: HFpEF-EF ≥ 50%, HFmrEF-EF 41–49%, and HFrEF-EF ≤ 40%.

The study sample consisted of 1144 patients: 496 (43.00%) men and 648 (57.00%) women. The HFpEF group consisted of 362 (32.00%) subjects, the HFmrEF group consisted of 263 (23.00%) subjects, and the HErEF group consisted of 519 (45.00%) subjects. A statistically significant distribution according to gender was observed between HFrEF vs. HFmrEF and HFpEF vs. HFrEF (*p* < 0.001). The majority of participants in the HFpEF and HFmrEF groups were women, whereas men predominated in the HFrEF group. There was a statistically significant age difference observed between HFpEF vs. HFrEF and HFrEF vs. HFmrEF (*p* < 0.001). Patients in the HFpEF and HFmrEF groups were older than those in the HFrEF group. The majority of patients had III functional class HF according to the New York Heart Association (NYHA) classification. Detailed results are shown in Table 1.

### 3.2. Distribution of Comorbidities in Study Groups

Patients with HF had a wide range of comorbidities that contributed to the onset and progression of the disease. The most common comorbidity was arterial hypertension (AH), which was present in 1101 of 1144 patients. The distribution of comorbidities varied between the three HFEF groups. Detailed results are shown in Table 2.

### 3.3. Laboratory Parameters Among Patients in Different HF Groups

When analyzing laboratory test results of patients in different HF groups, it was observed that HFrEF patients had significantly higher blood concentrations of hemoglobin (HGB), C-reactive protein (CRP), creatinine, aspartate aminotransferase (AST), alanine aminotransferase (ALT), alkaline phosphatase (ALP), gamma-glutamyl transferase (GGT), uric acid, and *n*-terminal pro-B-type natriuretic peptide (NT–ProBNP) compared to the HFpEF group. Additionally, significantly higher HGB, AST, and ALT levels were observed in the HFrEF group compared to the HFmrEF group. No significant differences were found in other laboratory parameters (Table 3).

### 3.4. Electrocardiographic Data in Different HF Droups

This study analyzed parameters of electrocardiograms (ECGs). The majority of subjects had chronic atrial fibrillation (AF), which was most common in the HFrEF group. Detailed results are presented in Table 4.

### 3.5. Echocardiographic Parameters Among Patients in Different HF Groups

When comparing the differences in echocardiographic parameters between the three groups, significantly larger values of left ventricular end-diastolic diameter (LVEDD), left ventricular mass index (LVMI), left atrial (LA) size and volume, and right ventricular (RV) and right atrial (RA) sizes were found in HFrEF patients when compared to those in the HFpEF group (*p* < 0.001). HFrEF patients were observed to have lower tricuspid annular plane systolic excursion (TAPSE) values than those in the HFmrEF and HFpEF groups (*p* < 0.001). Detailed results are described in Table 5.

### 3.6. Patient Outcomes in Different HF Groups

There were no statistically significant different outcomes (discharged home, deceased, transferred to a tertiary-level hospital, and transferred to nursing) in different HFEF groups. Detailed results are presented in Table 6.

When comparing the duration of in-hospital stay between different HF groups, a significant difference between the HFpEF and HFmrEF groups was found (*p* = 0.047). No significant differences were found between HFpEF and HFrEF (*p* = 0.289) or HFmrEF and HFrEF (*p* = 0.843) groups. The results are presented in Table 7.

## 4. Discussion

Our study revealed differences between the three HF groups, which were categorized based on LVEF values. In our study, which included 1144 patients, the overall prevalence of HFpEF was lower than that of HFrEF. Wang et al. evaluated a large patient cohort and reported a similar distribution between HF groups [8]. Similarly, Shiga et al. reported an overall HFpEF prevalence of 43% among 1245 patients with HF [9]. Moreover, our analysis confirmed that men were more frequently diagnosed with HFrEF than women, which is consistent with findings from other studies [10]. A study published in 2019 analyzed gender differences and found that most women belonged to the HFpEF group while most men belonged to the HFrEF group [11]. Similarly, our study observed the same trend in gender distribution.

Our study also analyzed the comorbidities of patients and their frequency distribution among the HFEF groups. Patients in the HFrEF group were statistically significantly more likely to have an ischemic etiology when compared both with HFpEF and HFmrEF groups. Patients in this group were also more likely to be diagnosed with dilated cardiomyopathy than patients in HFpEF and HFmrEF groups. In a comprehensive review of similarities and differences between HFpEF and HFrEF groups, published by Li and colleagues, it was noted that HFpEF patients were less likely to have ischemic etiology but had a higher burden of AH and AF [12]. However, in our study, AF was more common in the HFmrEF group when compared to HFpEF and HFrEF groups, whereas AH was more common in the HFpEF patients compared to those with HFrEF. The prevalence of AF between HF groups also varies in different literature sources. In a prospective analysis of 405 patients conducted by Xu et al., the prevalence of AF was higher in patients with HFpEF and HFmrEF compared to patients with HFrEF [13]. However, a study conducted by Scholten and colleagues reports different results. Among 5029 patients with HF, AF prevalence was highest in the HFpEF group and lowest in the HFmrEF group. The study also reported AH as the most commonly occurring comorbidity, affecting around three-quarters of HF patients, which aligns with our findings. However, in our cohort, the prevalence of hypertension was even higher, indicating a particularly heavy burden of this comorbidity. Our study also reported a higher rate of chronic kidney disease and diabetes mellitus in HF patients when compared to a study conducted by Scholten [14].

When comparing laboratory test results among the LVEF groups, hemoglobin concentration was significantly lower in the HFpEF group than in the HFrEF group. Similar findings were reported in a retrospective study conducted by Miller et al., who also found a higher prevalence of anemia in HFpEF patients [15]. Our study also revealed that patients with HFrEF had significantly higher blood concentrations of AST, ALT, ALP, and GGT compared to those with HFpEF. These findings may be attributed to hepatic congestion and hypoperfusion, both of which are common in HFrEF. Reduced LVEF leads to a decreased cardiac output and increased central venous pressure, thus contributing to liver dysfunction [16]. Furthermore, when comparing all three groups—HFpEF, HFmrEF, and HFrEF—NT-ProBNP levels were lowest in HFpEF patients and highest in those with HFrEF. A study conducted by Savarese and colleagues reported similar trends [17]. Elevated NT-proBNP levels are associated with increased risks of all-cause mortality, cardiovascular mortality, and HF hospitalization [18]. These findings underscore the importance of NT-proBNP as a prognostic marker in HF patients.

When analyzing the electrocardiographic parameters of HF patients, statistically significant differences were observed between the HFEF groups regarding the presence of bundle branch block (BBB), chronic AF, and ventricular extrasystoles. Patients in the HFrEF group were statistically significantly more likely to be diagnosed with BBB than HFpEF and HFmrEF patients. HFrEF patients were also more likely to experience ventricular extrasystoles when compared to both HFpEF and HFmrEF groups. Chronic AF was most common among the patients in the HFmrEF group. However, in a study of a long-term registry of the European Society of Cardiology, the findings of the prevalence of AF in HF patients showed different trends, with the prevalence being 27% in HFrEF, 29% in HFmrEF, and 39% in the HFpEF group [19]. Sebastian Rosch et al. also found that AF was more frequently present on admission in the LVEF 50% to 60% cohort [20]. Nikolaidou et al. also found that AF was more common in HFpEF patients compared to those with HFrEF [21].

When comparing the differences in echocardiographic parameters between the groups, significantly larger values of LVEDD, LVMI, LA size and volume, and RV and RA sizes were found in HFrEF patients when compared to those in both HFpEF and HFmrEF groups. Additionally, HFrEF patients were observed to have lower TAPSE values than those in the HFpEF group, suggesting that patients with HFrEF have a significant remodeling of both left and right ventricles. In a study conducted by Harada and colleagues, which evaluated functional and morphological echocardiographic parameters of HFpEF and HFrEF groups, LVEDD, LVMI, and LA volume values were found to be significantly higher in the HFrEF group compared to the HFpEF group as well [22]. Palazzuoli and colleagues conducted a study and found different results. The average RV size value in the HFpEF group was 43 mm, compared to 39 mm in the HFrEF group [23]. In a similar study by Bosch et al., which investigated and compared the prevalence of RV dysfunction between the HFpEF and HFrEF groups, TAPSE values were found to be significantly lower in the HFrEF group, similarly to our findings [24].

In our study, no statistically significant differences in patient outcomes were observed between the three LVEF groups. The percentages of patients discharged home, transferred to tertiary-level cardiology hospitals, or to palliative care institutions, as well as in-hospital mortality rates, were similar across all HF groups (Table 6). Although the mean duration of hospitalization was similar across all groups (ranging from 9 to 10 days), a statistically significant difference was observed between the HFpEF and HFmrEF groups (Table 7). Previous studies have demonstrated varying outcomes when comparing different HF groups. Data from the Atherosclerosis Risk in Communities study indicated that patients with HFpEF had a higher average number of comorbidities than those with HFrEF, which was also associated with an increased mortality risk [25]. However, a prospective multicenter study reported different findings, showing that HFpEF patients had a lower risk of mortality compared to those with HFrEF [26]. A study conducted by Olchanski and colleagues also found that patients with HFrEF had a longer median length of in-hospital duration compared to those with HFpEF [27], which differs from our findings.

The results of our study may support and strengthen patient care strategies by emphasizing the importance of individualized management across different HF groups. Recognizing the distinct clinical, laboratory, and echocardiographic profiles of HFrEF, HFmrEF, and HFpEF can help clinicians better identify high-risk patients, improve prognostic evaluation, and tailor therapy to individual patient needs. For example, a higher burden of right ventricular dysfunction and atrial enlargement in HFrEF patients may prompt closer monitoring of right heart function and early consideration of advanced therapies. In HFpEF, the predominance of comorbidities like hypertension and AF supports the continued focus on managing these conditions in the absence of effective disease-modifying therapies. These findings support a phenotype-guided approach to HF that moves beyond ejection fraction alone and promotes individualized follow-up, multidisciplinary care, and therapy tailored towards each HF subtype.

This study also had several limitations. It was a retrospective single-center study, which may limit the generalizability of the findings and introduce selection bias. Only patients hospitalized in the Cardiology Department of our hospital were included, potentially excluding individuals with milder HF symptoms who were managed in outpatient settings or those treated in other departments or institutions. The collection of the data was limited to the in-hospital period, preventing evaluation of long-term outcomes such as post-discharge mortality and rehospitalization rates across HF groups. Additionally, data on medication use and post-discharge management strategies was not included, which may have influenced the patient outcomes.

Future prospective, multicenter studies with extended follow-up are needed to validate our findings, assess the predictive role of right heart echocardiographic parameters across HF phenotypes, and evaluate whether phenotype-guided treatment strategies can improve long-term clinical outcomes.

## 5. Conclusions

HFrEF patients were younger and more frequently male compared to HFpEF and HFmrEF patients. Arterial hypertension and atrial fibrillation were more frequent in the HFpEF group, whereas ischemic etiology and dilated cardiomyopathy were more frequently observed in HFrEF patients. Echocardiographic analysis showed that HFrEF patients had larger chamber sizes, signs of LV remodeling, and RV dysfunction when compared to HFpEF patients. The study results also confirmed that NT-proBNP concentration was the lowest in the HFpEF group and the highest in the HFrEF group, highlighting the importance of this biomarker in the classification of HF and its prognostic value.

Future research is needed to evaluate the prognostic value of such echocardiographic parameters as TAPSE and right atrial size in different HF phenotypes. Future studies could also evaluate long-term outcomes of the patients such as mortality and rehospitalization rates across different HF phenotypes, extending beyond the in-hospital setting. This could provide a more accurate and comprehensive understanding of mortality and rehospitalization patterns across the different HF phenotypes. Moreover, additional research might also explore whether phenotype-guided treatment strategies improve long-term outcomes and patient-specific management in different HF groups.

## Figures and Tables

**Table 1:** Patients’ characteristics.

Characteristics	Total in the Study Sample	HFpEF	HFmrEF	HFrEF	*p* Value
Male, *n* (%)	496 (43.00%)	102 (21.00%)	84 (17.00%)	310 (62.00%)	HFpEF vs. HFmrEF *p* = 0.177 HFrEF vs. HFmrEF ***p* < 0.001** HFpEF vs. HFrEF ***p* < 0.001**
Female, *n* (%)	648 (57.00%)	260 (40.00%)	179 (28.00%)	209 (32.00%)	
Age (years)					
Age, median (min-max)	81 (29–102)	84 (43–102)	84 (49–100)	77 (29–100)	HFrEF vs. HFpEF ***p* < 0.001** HFrEF vs. HFmrEF ***p* < 0.001** HFpEF vs. HFmrEF *p* = 0.525
NYHA Classes					
I, *n* (%)	1 (0.10%)	1 (0.28%)	0 (0.00%)	0 (0.00%)	HFpEF vs. HFrEF ***p* < 0.001** HFpEF vs. HFmrEF ***p* < 0.001** HFrEF vs. HFmrEF *p* = 0.269
II, *n* (%)	28 (2.40%)	18 (4.97%)	4 (1.52%)	6 (1.16%)	
III, *n* (%)	985 (86.10%)	318 (87.85%)	231 (87.83%)	436 (84.00%)	
IV, *n* (%)	89 (7.80%)	8 (2.20%)	21 (87.83%)	60 (11.56%)	

HFpEF—heart failure with preserved ejection fraction; HFmrEF—heart failure with mildly reduced ejection fraction; HFrEF—heart failure with reduced ejection fraction; HF—heart failure; NYHA—New York Heart Association (NYHA) classification.

**Table 2:** Distribution of comorbidities in different HF groups.

Comorbidities	Total in the Study Sample	HFEF Groups			*p* Value
		HFpEF	HFmrEF	HFrEF	
Diabetes mellitus, *n* (%)	301 (26.00%)	87 (24.00%)	79 (30.00%)	135 (26.00%)	*p* = 0.237
Arterial hypertension, *n* (%)	1101 (96.00%)	356 (98.34%)	259 (98.48%)	486 (93.64%)	HFpEF vs. HFmrEF *p* = 0.893 HFrEF vs. HFmrEF ***p* = 0.003** HFpEF vs. HFrEF ***p* < 0.001**
Chronic kidney disease, *n* (%)	438 (38.00%)	120 (33.15%)	117 (44.49%)	201 (38.73%)	*p* = 0.015
Ischemic etiology, *n* (%)	355 (31.00%)	60 (16.57%)	82 (31.18%)	213 (41.00%)	HFpEF vs. HFmrEF ***p* < 0.001** HFrEF vs. HFmrEF ***p* = 0.007** HFpEF vs. HFrEF ***p* < 0.001**
Dilated cardiomyopathy, *n* (%)	30 (2.60%)	1 (0.28%)	2 (0.76%)	27 (5.20%)	HFpEF vs. HFmrEF *p* < 0.387 HFrEF vs. HFmrEF ***p* = 0.002** HFpEF vs. HFrEF ***p* < 0.001**
Atrial fibrillation, *n* (%)	762 (66.60%)	225 (62.15%)	196 (74.52%)	341 (65.70%)	HFpEF vs. HFmrEF ***p* = 0.001** HFrEF vs. HFmrEF ***p* = 0.012** HFpEF vs. HFrEF *p* = 0.280
Pulmonary disease (asthma, previous embolism, COPD), *n* (%)	166 (14.50%)	48 (13.26%)	37 (14.00%)	81 (15.60%)	*p* = 0.594
Presence of infection during hospitalization, *n* (%)	375 (33.00%)	114 (31.49%)	88 (33.46%)	173 (33.33%)	*p* = 0.819

HFpEF—heart failure with preserved ejection fraction; HFmrEF—heart failure with mildly reduced ejection fraction; HFrEF—heart failure with reduced ejection fraction; HF—heart failure; COPD—chronic obstructive pulmonary disease.

**Table 3:** Laboratory data of patients in different HF groups.

Laboratory Results	HFEF Groups			*p*-Values
	HFpEF	HFmrEF	HFrEF	
HGB (g/L)	119.70 ± 20.06	120.19 ± 22.34	127.54 ± 23.16	HFpEF vs. HFrEF ***p* < 0.001** HFpEF vs. HFmrEF *p* = 0.960 HFrEF vs. HFmrEF ***p* < 0.001**
CRP (mg/L), median (min–max)	7.64 (0.60–261.56)	7.93 (0.60–220.08)	9.50 (0.60–234.00)	HFpEF vs. HFrEF ***p* = 0.009** HFpEF vs. HFmrEF *p* = 0.302 HFrEF vs. HFmrEF *p* = 0.208
WBC (×10^9^/L), median (min–max)	7.83 (2.85–23.17)	7.54 (2.21–31.22)	7.60 (2.81–23.48)	HFpEF vs. HFrEF *p* = 0.970 HFpEF vs. HFmrEF *p* = 0.730 HFrEF vs. HFmrEF *p* = 0.781
PLT (×10^9^/L), median (min–max)	216.00 (60.00–854.00)	208.00 (99.00–682.00)	209.00 (69.00–802.00)	HFpEF vs. HFrEF *p* = 0.199 HFpEF vs. HFmrEF *p* = 0.303 HFrEF vs. HFmrEF *p* = 0.920
Sodium (mmol/L), median (min–max)	139.60 (112.30–158.20)	139.60 (117.20–160.00)	139.10 (109.80–151.20)	HFpEF vs. HFrEF *p* = 0.246 HFpEF vs. HFmrEF *p* = 0.283 HFrEF vs. HFmrEF *p* = 0.028
Potassium (mmol/L), median (min–max)	4.41 (1.61–7.53)	4.46 (2.60–6.06)	4.40 (2.47–7.43)	HFpEF vs. HFrEF *p* = 0.967 HFpEF vs. HFmrEF *p* = 0.399 HFrEF vs. HFmrEF *p* = 0.347
Creatinine (µmol/L), median (min–max)	93.44 (23.87–388.25)	96.78 (44.11–289.70)	105.48 (26.53–427.20)	HFpEF vs. HFrEF ***p* < 0.001** HFpEF vs. HFmrEF *p* = 0.352 HFrEF vs. HFmrEF ***p* = 0.029**
Glucose (mmol/L), median (min–max)	6.67 (2.73–25.87)	6.52 (2.83–20.55)	6.68 (3.13–28.40)	HFpEF vs. HFrEF *p* = 0.139 HFpEF vs. HFmrEF *p* = 0.098 HFrEF vs. HFmrEF *p* = 0.681
AST (U/L), median (min–max)	22.05 (9.50–221.20)	22.50 (9.40–106.30)	25.90 (7.40–3216.70)	HFpEF vs. HFrEF ***p* = 0.004** HFpEF vs. HFmrEF *p* = 0.921 HFrEF vs. HFmrEF ***p* = 0.008**
ALT (U/L), median (min–max)	17.25 (3.50–248.00)	16.60 (3.00–109.90)	20.90 (4.50–2386.00)	HFpEF vs. HFrEF ***p* = 0.015** HFpEF vs. HFmrEF *p* = 0.987 HFrEF vs. HFmrEF ***p* = 0.017**
ALP (U/L), median (min–max)	85.32 (32.05–224.01)	94.58 (14.80 –584.38)	98.07 (35.58–1402.00)	HFpEF vs. HFrEF ***p* = 0.007** HFpEF vs. HFmrEF *p* = 0.261 HFrEF vs. HFmrEF *p* = 0.146
GGT (U/L), median (min–max)	37.92 (5.69–416.30)	51.77 (7.13–345.86)	77.94 (9.00–1125.59)	HFpEF vs. HFrEF ***p* < 0.001** HFpEF vs. HFmrEF *p* = 0.028 HFrEF vs. HFmrEF *p* = 0.024
Urea (mmol/L), median (min–max)	9.30 (4.30–172.20)	11.93 (2.45–36.39)	10.69 (3.21–110.80)	HFpEF vs. HFrEF *p* = 0.143 HFpEF vs. HFmrEF *p* = 0.020 HFrEF vs. HFmrEF *p* = 0.230
Uric acid (µmol/L), median (min–max)	412.22 (172.80–916.40)	444.00 (235.62–922.77)	481.85 (219.07–921.97)	HFpEF vs. HFrEF ***p* = 0.011** HFpEF vs. HFmrEF *p* = 0.436 HFrEF vs. HFmrEF *p* = 0.118
TP (g/L)	61.31 ± 7.32	60.80 ± 7.58	60.06 ± 6.75	HFpEF vs. HFrEF *p* = 0.259 HFpEF vs. HFmrEF *p* = 0.837 HFrEF vs. HFmrEF *p* = 0.641
Albumin (g/L), median (min–max)	37.67 (16.14–49.88)	36.38 (17.17–51.20)	37.01 (17.61–59.58)	HFpEF vs. HFrEF *p* = 0.157 HFpEF vs. HFmrEF *p* = 0.068 HFrEF vs. HFmrEF *p* = 0.587
NT–ProBNP (pg/mL), median (min–max)	2411.00 (73.39–35,000.00)	3943.00 (155.00–35,000.00)	6487.00 (41.72–35,000.00)	HFpEF vs. HFrEF ***p* < 0.001 ** HFpEF vs. HFmrEF ***p* < 0.001** HFrEF vs. HFmrEF ***p* < 0.001**

HFpEF—heart failure with preserved ejection fraction; HFmrEF—heart failure with mildly reduced ejection fraction; HFrEF—heart failure with reduced ejection fraction; HGB—hemoglobin; CRP—C-reactive protein; WBC—white blood cell count; PLT—platelet; AST—aspartate aminotransferase; ALT—alanine aminotransferase; ALP—alkaline phosphatase; GGT—gamma-glutamyl transferase; TP—total protein; NT–ProBNP—*n*-terminal pro-B-type natriuretic peptide.

**Table 4:** Electrocardiographic parameters of patients in different HF groups.

ECG Parameters	Total in the Study Sample	HFEF Groups			*p* Value
		HFpEF	HFmrEF	HFrEF	
Bundle branch block, *n* (%)	293 (26.00%)	71 (19.61%)	49 (18.63%)	173 (33.30%)	HFpEF vs. HFrEF ***p* < 0.001** HFpEF vs. HFmrEF *p* = 0.758 HFrEF vs. HFmrEF ***p* < 0.001**
Chronic AF, *n* (%)	565 (56.50%)	159 (43.92%)	153 (58.17%)	253 (48.75%)	HFpEF vs. HFmrEF ***p* < 0.001** HFrEF vs. HFmrEF ***p* = 0.013** HFpEF vs. HFrEF *p* = 0.158
Persistent AF, *n* (%)	167 (14.60%)	55 (15.19%)	37 (14.00%)	75 (14.45%)	*p* = 0.918
Paroxysmal AF, *n* (%)	22 (1.92%)	10 (2.76%)	5 (1.90%)	7 (1.35%)	*p* = 0.323
Ventricular extrasystoles, *n* (%)	208 (18.00%)	51 (14.09%)	38 (14.45%)	119 (22.93%)	HFpEF vs. HFmrEF *p* = 0.899 HFrEF vs. HFmrEF ***p* = 0.005** HFpEF vs. HFrEF ***p* = 0.001**
Conduction disorders, *n* (%)	44 (3.80%)	20 (5.52%)	5 (1.90%)	19 (3.66%)	*p* = 0.064

HFpEF—heart failure with preserved ejection fraction; HFmrEF—heart failure with mildly reduced ejection fraction; HFrEF—heart failure with reduced ejection fraction; HF—heart failure; AF—atrial fibrillation; ECG—electrocardiogram.

**Table 5:** Echocardiographic parameters of patients in different HF groups.

Echocardiographic Parameters	HFEF Groups			*p*-Values
	HFpEF	HFmrEF	HFrEF	
LVEDD (mm) median (min–max)	46.50 (26.00–68.00)	48.00 (32.00–71.00)	55.00 (30.00–92.00)	HFpEF vs. HFrEF ***p* < 0.001** HFpEF vs. HFmrEF *p* = 0.271 HFrEF vs. HFmrEF ***p* < 0.001**
LVMI (g/m^2^), median (min–max)	102.13 (37.55–175.30)	108.88 (56.91–228.89)	131.42 (60.03–267.15)	HFpEF vs. HFrEF ***p* < 0.001** HFpEF vs. HFmrEF ***p* = 0.002** HFrEF vs. HFmrEF ***p* < 0.001**
LA size (mm), median (min–max)	46.00 (27.00–98.00)	48.00 (29.00–80.00)	50.00 (28.00–81.00)	HFpEF vs. HFrEF ***p* < 0.001** HFpEF vs. HFmrEF ***p* < 0.001** HFrEF vs. HFmrEF ***p* < 0.001**
LA volume (mL/m^2^), median (min–max)	85.50 (29.00–656.00)	96.00 (28.00–315.00)	110.00 (12.00–468.00)	HFpEF vs. HFrEF ***p* < 0.001** HFpEF vs. HFmrEF ***p* < 0.001** HFrEF vs. HFmrEF ***p* < 0.001**
RV size (mm), median (min–max)	37.00 (22.00–67.00)	40.00 (27.00–62.00)	42.00 (23.00–75.00)	HFpEF vs. HFrEF ***p* < 0.001** HFpEF vs. HFmrEF ***p* < 0.001** HFrEF vs. HFmrEF ***p* < 0.001**
RA size (mm), median (min–max)	41.60 (24.00–73.00)	44.00 (31.00–68.00)	48.30 (26.00–77.00)	HFpEF vs. HFrEF ***p* < 0.001** HFpEF vs. HFmrEF ***p* < 0.001** HFrEF vs. HFmrEF ***p* < 0.001**
TAPSE (mm), median (min–max)	20.00 (11.00–38.00)	17.00 (6.30–38.00)	15.50 (6.00–33.00)	HFpEF vs. HFrEF ***p* < 0.001** HFpEF vs. HFmrEF ***p* < 0.001** HFrEF vs. HFmrEF *p* = 0.054

HFpEF—heart failure with preserved ejection fraction; HFmrEF—heart failure with mildly reduced ejection fraction; HFrEF—heart failure with reduced ejection fraction; LVEDD—left ventricular end-diastolic diameter; LVMI—left ventricular mass index; LA—left atrium; RV—right ventricle; RA—right atrium; TAPSE—tricuspid annular plane systolic excursion.

**Table 6:** Patient outcomes in different HF groups.

Outcomes	Total in the Study Sample	HFEF Groups			*p* Value
		HFpEF	HFmrEF	HFrEF	
Discharged home, *n* (%)	938 (82.00%)	302 (83.43%)	217 (82.50%)	419 (80.73%)	*p* = 0.548
In-hospital death, *n* (%)	61 (5.00%)	13 (3.59%)	17 (6.46%)	31 (5.97%)	*p* = 0.195
Transferred to a tertiary-care cardiology hospital, *n* (%)	86 (7.50%)	30 (8.29%)	13 (4.94%)	43 (8.29%)	*p* = 0.197
Transferred to palliative care hospital, *n* (%)	56 (4.60%)	15 (4.14%)	14 (5.32%)	24 (4.62%)	*p* = 0.789

HFpEF—heart failure with preserved ejection fraction; HFmrEF—heart failure with mildly reduced ejection fraction; HFrEF—heart failure with reduced ejection fraction; HF—heart failure.

**Table 7:** Duration of in-hospital stay in different HF groups.

	HFEF Groups			*p*-Values
	HFpEF	HFmrEF	HFrEF	
Duration of in-hospital stay (days), mean ± SD	9.32 ± 4.88	10.02 ± 4.48	9.79 ± 5.03	HFpEF vs. HFrEF *p* = 0.289 HFpEF vs. HFmrEF ***p* = 0.047** HFrEF vs. HFmrEF *p* = 0.843

HFpEF—heart failure with preserved ejection fraction; HFmrEF—heart failure with mildly reduced ejection fraction; HFrEF—heart failure with reduced ejection fraction; HF—heart failure.

## Data Availability

The data presented in this study are available on request from the corresponding author due to privacy and legal reasons.

## Abbreviations

The following abbreviations are used in this manuscript:

HF	Heart failure
LVEF	Left ventricular ejection fraction
HFrEF	Heart failure with reduced ejection fraction
HFmrEF	Heart failure with mildly reduced ejection fraction
HFpEF	Heart failure with preserved ejection fraction
NYHA	New York Heart Association
AH	Arterial hypertension
COPD	Chronic obstructive pulmonary disease
HGB	Hemoglobin
CRP	C-reactive protein
AST	Aspartate aminotransferase
ALT	Alanine aminotransferase
ALP	Alkaline phosphatase
GGT	Gamma-glutamyl transferase
NT–ProBNP	*n*-terminal pro-B-type natriuretic peptide
WBC	White blood cell count
PLT	Platelet
TP	Total protein
ECG	Electrocardiogram
AF	Atrial fibrillation
LVEDD	Left ventricular end-diastolic diameter
LVMI	Left ventricular mass index
LA	Left atrium
RV	Right ventricle
RA	Right atrium
TAPSE	Tricuspid annular plane systolic excursion
BBB	Bundle branch block
